# Effects of high-intensity interval training program on pituartry function in basketball players: a randomized controlled trial

**DOI:** 10.3389/fphys.2023.1219780

**Published:** 2023-11-22

**Authors:** Recep Soslu, Abdullah Uysal, Meltem Devrilmez, İsmail Can Çuvalcıoğlu, Ali Ahmet Doğan, Sülbiye Karaburgu, Murat Taş

**Affiliations:** ^1^ Department of Coaching Education, Faculty of Sports Sciences, Karamanoglu Mehmetbey University, Karaman, Türkiye; ^2^ Institue of Healt Sciences, Karamanoglu Mehmetbey University, Karaman, Türkiye; ^3^ Faculty of Sports Sciences, Kırıkkale University, Kırıkkale, Türkiye; ^4^ Department of Endocrinolgy, Medical Faculty, Karamanoglu Mehmetbey University, Karaman, Türkiye; ^5^ Faculty of Sports Sciences, Celal Bayar University, Manisa, Türkiye

**Keywords:** cortisol, basketball, interval training, pituitary function, HIIT

## Abstract

The aim of the study was to determine how the short high-interval training program affects cortisol levels in basketball players. A total of 27 male basketball players volunteered for the study and were randomly assigned to one of two groups: Control Group (CG) (*n* = 13, mean age; 20,56 ± 1,45 years, mean height; 184,53 ± 6,65 cm) and Experimental Group (EG) (*n* = 14, mean age; 20,71 ± 2,12 years, mean height; 86,51 ± 8,21 cm). The experimental group received a 7-week interval training program. Blood samples were taken at the beginning and end of the study. Cortisol, Adrenocorticotropic Hormone, Triiodothyronine, Tetraiodothyronine, Parathyroid Hormone, Thyroid Stimulating Hormone, Insulin, and Glucose levels were measured of the EG and CG. To test the differences between groups and compare the effects of pre and post-intervention, a two-way ANOVA for repeated measures was used. Consequently, the post-test levels of TSH, PTH, and ACTH in the exercise group showed a significant difference when compared to the pre-test values (*p* = 0.000). Moreover, the post-test levels of Glucose, Cholesterol, Triglyceride, HDL, and Mg in the exercise group showed statistical significance when compared to the pre-test values (*p* = 0.000). Significant differences were seen in the post-test PTH and ACTH levels of the control group when compared to the pre-test values (*p* = 0.000). Furthermore, the exercise group showed significant differences in post-test values for HDL and Cholesterol compared to the pre-test (*p* = 0.000). In addition, when comparing the post-test values and pre-test values of both the exercise group and the control group, it was found that all parameters, with the exception of Ca, exhibited substantial differences in favor of the exercise group. It may be claimed that the implementation of interval training has favorable outcomes on pituitary function parameters. Additionally, the regulation of energy consumption during exercise is favourably influenced, along with the reduction of physiological stress resulting from prolonged exercise.

## Introduction

The endocrine system exhibits functionality in virtually all tissues throughout the body. The physiological process of preparing the body for physical activity, as well as its ability to adapt to different stressors, holds significant importance in many bodily adaptations. The endocrine system plays a crucial role in facilitating exercise performance, promoting long-term adaptations to training in the human body, and eventually enhancing exercise and sports performance. Cortisol is a glucocorticoid hormone released by the adrenal cortex in response to stress, and it reaches its peak plasma levels 1 h before waking up (Jones and Gwenin, 2021). Glucocorticoids influence gluconeogenesis, the immune system, and the central nervous system (Hackney, 2006). It also hastens the synthesis of glucose from protein and fat in the liver (gluconeogenesis), resulting in an increase in the amount of free fatty acids in the blood (Hall and Guyton, 2013). Cortisol is buffered by various tissues in the body, including skeletal muscle, adipose tissue, and the liver, after it is released (Hill et al., 2008). For example, in addition to breaking down proteins into amino acids in skeletal muscle, it can hydrolyze triglycerides in adipose tissue into free triglycerides (Hall and Guyton, 2013), whereas high levels of circulating cortisol can stimulate liver gluconeogenesis by providing additional carbohydrates required for energy production. Hill et al. (2008). This effect has a positive impact on critical physiological processes that aid in increasing exercise capacity. Furthermore, cortisol activates the body’s defense mechanism against any stress (such as intense exercise, hunger, and anxiety) (Silverman and Deuster, 2014; Philippou et al., 2021). One of the leading causes of these stresses is physical activity. It is because aerobic systems and glycolysis provide the energy needed during exercise (Tanriverdi et al., 2008). As a result, the intensity of exercise (the amount of cortisol produced and the increased energy deficit) raises the amount of cortisol produced (Roy and Parker, 2007). The adrenocorticotropic hormone, commonly referred to as adrenocorticotropic hormone (ACTH), and cortisol are both indicators of anabolic and catabolic balance, and their levels often increase in response to exhaustion. Both stress and exhaustion cause a rise in the blood levels of the hormone ACTH, as well as cortisol (Hill et al., 2008).

Moreover is well-known that hormones produced by the thyroid increase both the resting metabolic rate and the rate at which proteins are synthesized. Thyroid hormones (Triiodothyronine (T3)), Tetraiodothyronine (T4)) also have an influence on the catecholamine effect. However it is not very clear how thyroid hormones will respond to the various forms of exercise. Both the loss of body fat and the increase in the amount of energy used during endurance training are processes that are largely regulated by thyroid hormones (Ciloglu et al., 2005). Therefore Aerobic exercise increases levels of T3 and T4 in a manner that is dependent on the intensity of the exercise. The handling of blood glucose is one of the most obvious advantages of aerobic exercise. Due to the fact that aerobic exercise is a stressor that reduces blood glucose, which is crucial for body cells. By increasing GLUT4, also known as the “muscle glucose carrier protein,” regular exercise reduces insulin resistance and enhances glucose uptake into the muscle (Kraemer et al., 1999). After exercise, blood glucose levels decrease and insulin secretion increases between 24 and 72 h. Studies have also shown that the blood glucose reduction is related to the volume and duration of exercise, and that similar benefits might persist for 5–7 days following exercise (Galbo et al., 2007; O'Gorman et al., 2006).

Interval training is the systematic application of many exercise series with a certain interval, rest and different loads (intensity) (Gibala, 2021). The maximum heart rate of the athlete is reduced to 80%–90%, and rest intervals are formed by lowering the maximum heart rate to 40%–50% (Roy, 2013). The intensity and duration of the intervals determine the training duration (Gaesser and Angadi, 2011; Roy, 2013). It should be noted that resting during interval training is not the same as resting completely. The reasons for this include increased endurance, improved performance capacity, and fatigue resistance (Arazi et al., 2014; Grace et al., 2017). The body’s responses to stress during interval training include skeletal muscle damage, increased energy and oxygen demand, and decreased energy stores (Buchheit and Laursen, 2013). Thus, ınterval training, which is largely based on aerobic energy capacity, and particularly middle and long distance running, has long been used by athletes (Gibala, 2021). Considered in this context, the amount of cortisol produced during high-intensity exercise affects skeletal muscle function significantly (Sheffield-Moore and Urban, 2004). There is an associated link between high-intensity exercise and higher cortisol levels (Hill et al., 2008; Skoluda et al., 2012). Hill et al. discovered that moderate to high intensity (60%, 80%) exercise raises circulating cortisol levels. These increases are thought to be mediated by a combination of hemoconcentration and increased secretion stimuli (i.e., ACTH) in the hypothalamic-pituitary-adrenocortical axis (Hill et al., 2008). When the literature is reviewed, it is discovered that the duration, intensity, and circadian rhythm of exercise influence the change in plasma cortisol levels (Gabriel and Zierath, 2019; Hill and Chtourou, 2020).

Long-term exercise has been shown to increase cortisol levels, but short-term low-intensity exercise has not been shown to change cortisol levels or only slightly (Tsai et al., 2014; Wilk et al., 2020; Gordon et al., 2021). Understanding the body’s responses is crucial to comprehending the entire stress placed on basketball players during match or training in addition to the above basketball needs. This is an effective tool for determining whether or not an basketball player has overtrained, as well as for preventing excessive psychophysical effect caused by competition (Martínez et al., 2010). The endocrine system is strongly impacted by the increased energy needs experienced by basketball players during competition or training. During exercise, there is an equal rise in glucagon concentrations and a decrease in insulin concentrations, as glucose and free fatty acids are both essential sources of energy. Providing consistent blood glucose concentrations is considered a crucial aspect of exercise. Experiencing a loss of balance or tripping when engaging in exercise. The maintenance of stable blood glucose levels is facilitated by the presence of insulin and elevated glucagon concentrations (Braun and Horton, 2001). In addition, elevated blood lactate levels imply that fast glycolysis contributes significantly to players’ energy needs, but HR responses give an imprecise understanding of the use of aerobic energy sources (Stojanović et al., 2018). Moreover, it is well-established that thyroid hormones have the capacity to enhance the basal metabolic rate, protein synthesis, and the impact of catecholamines. But the specific effect of different types of vigorous exercise on thyroid hormone levels remains uncertain. According to one study, endurance exercise led to a considerable drop in T3 hormone levels while simultaneously leading to a significant rise in T4 levels (Ciloglu et al., 2005).

The negative effects caused by incorrect or over-training during the basketball season persist throughout the duration of the season. Even while training and competition during the season are linked to stress and changes in the anabolic/catabolic balance, maintaining permanent management of anabolic and catabolic changes in addition to sensible planning for training is extremely critical. It is crucial to take into consideration the players’ physical situation as well as their level of recovery. There are studies in the literature that look at how exercise affects the cortisol. The number of studies on the effect of interval training on the pituarty function, however, is insufficient. The study’s goal, from this perspective, was to determine how the high-interval training program affects pituarty functions in basketball players.

## Materials and methods

### Participant

The study was designed as an experimental research model, which includes administering the performance parameters to be measured under specific rules and conditions, measuring the athletes’ responses, and analyzing the results obtained by comparing them. A total of 27 male semi-professionals basketball players volunteered to participate in the study (Table 1). Athletes were randomly assigned to one of two groups in the study: control and experimental (CG and EG, respectively). The athletes were informed about the study and advised not to take any medication that would impair their performance during the test measurements. According to reports, none of the athletes had pituitary disorders. Furthermore, none of them were taking any medication for pituitary disorders. The anatomical structures of all athletes participating in the study were healthy, and they had no injury-induced medical and orthopedic problems the lower/upper extremities. Athletes were included in the study after giving their (free and informed) consent in accordance with the Declaration of Helsinki’s ethical principles (2008). The Non-Clinical Ethics Committee of Karamanolu Mehmetbey University provided ethical approval (Decision No: 08-2022-07).

**TABLE 1:** Athletes’ demographic characteristics.

	E.G (N = 14) pre test	E.G (N = 14) post test	K.G (N = 13) pre test	K.G (N = 13) post test
	X ± Sd	X ± Sd	X ± Sd	X ± Sd
Age (yr)	20,71 ± 2,12	Null	20,56 ± 1,45	Null
H (cm)	186,51 ± 8,21	Null	184,53 ± 6,65	Null
BW (kg)	75,96 ± 4,32	72,11 ± 2,75	74,85 ± 3,53	73,5 ± 2,53
BMI (kg/m2)	21,9 ± 1,85	20,8 ± 1,05	22 ± 2,45	21,8 ± 1,95
SE (yr)	8,76 ± 3,25	Null	8,56 ± 3,63	Null

EG, Experimental Group; CG, Control Group; H, height; BW, body weight; BMI, body mass index; SE, sports experience.

### Experimental procedure

Each athlete underwent a medical examination as well as anthropometric measurements. A stadiometer was used to determine the athletes’ body weight and height (SECA-Mod. 220, Seca GmbH&Co. KG., Hamburg, Germany). Athletes performed a maximum grade field test until they felt tired before and after the 7-week training program to determine their Maximal Aerobic Velocity (MAV). The maximum test was run on a 200-m tartan track marked with cones. Blue cones were placed at 50 m intervals along the track (inside the first line), while red cones were placed 2 m behind the blue cones. The running speed was set by the specialist, and when the athlete had to pass by a cone to maintain a constant speed for each test phase, a short sound was emitted. For each sound, subjects had to be within 2 m of the blue cones. During the test, the subjects were required to stand three times in a row behind a red cone, or until they became fatigued and stopped exercising. The speed was initially set at 8 km/h and increased by 1 km/h every 2 min. The maximum aerobic velocity was determined by the speed in the last completed phase (Ben Abderrahman et al., 2013). The test was carried out in the morning, 3–4 h after a standard breakfast. Subjects were allowed to drink water between the end of the standardized breakfast and the start of the test.

### Biochemical measurements

Two blood samples were taken from the experimental and control groups at the beginning and end of the training program. The last blood samples were taken 2 days after the training program came to an end (such a process was done to avoid any acute weariness that may have resulted from the most recent exercise). Blood samples were taken in the morning (7:00-8:00) because the amount of hormone released during the day is highest in the morning. Blood was drawn from the antecubital vein (5 ml). Blood samples were collected into ethylenediaminetetraacetic acid (EDTA) containing tubes and centrifuged at 1,000 rpm for 20 min at 4°C. Obtained plasma was aliquoted and stored at −20°C until later analyses. Commercially available human ELISA kits were used in duplicate to measure plasma Cortisol (morning sensitivity: 20–800 ng/dl; RE52061, IBL International, Germany), Adrenocorticotropic (ACTH) (sensitivity: 7.2–63.3 pg/mL; RE53081 IBL International, Germany), Triiodothyronine (T3) (sensitivity: 0.4–18 pg/ml; RE55231 IBL International, Germany), Tetraiodothyronine (T4) (sensitivity: 0.3–7 ng/ml RE55241 IBL International, Germany), Parathyroid Hormone (PTH) (sensitivity: 15–65 pg/ml; NM59041 IBL International, Germany), Thyroid Stimulating Hormone (TSH) (sensitivity: 0.35–4.5 mU/ml; RE55221 IBL International, Germany), Insulin (sensitivity: 70–126 mg/dL; RE53171 IBL International, Germany) according to the procedures provided by the Manufacturer. Following a thorough physical examination that included the measurement of arterial blood pressure and pulse, laboratory testing was performed. The Biochemistry Laboratory (Karamanolu Mehmetbey University Biochemistry Laboratory, Karaman, Turkey) performed a series of blood tests to evaluate liver function, including lipid profile (total cholesterol, high-density lipoprotein cholesterol, and triglyceride levels), fasting glucose level, calcium level, magnesium level, and albumin level. All measurements were performed on the same day and reported in respective SI units.

### Intervention protocols

The pre-season period included two periods of training. The athletes were incorporated into the high-intensity interval training (HIIT) program, which included both technical and tactical training components. The athletes’ HIIT training involved doing technical and tactical training sessions within the training facility, rather than outside. The training program was executed by a team of highly qualified and experienced staff members, who closely monitored the players’ participation in the training sessions. The athletes engaged in the HIIT program on a tri-weekly basis, namely on Mondays, Wednesdays, and Fridays, over a duration of 7 weeks. This resulted in a total of 21 HIIT sessions, as indicated in Table 2. The HIIT sessions were scheduled with a minimum of 48 h of rest in between to provide sufficient recovery time. Throughout each session, measurements of temperature, humidity, and wind speed were consistently recorded. The HIIT sessions were comprised only of intermittent workouts coupled with active recovery periods, specifically designed for athletes. Each session included of three distinct periods. Prior to the sessions, a standardized warm-up procedure was conducted. This warm-up procedure entailed a 15-min continuous running activity, followed by a 5-min session of stretching exercises. Additionally, the warm-up procedure included 5 brief bursts of accelerations on the track. In each HIT session conducted on the track, a single participant was assigned to each lane. The examiner established predetermined lengths for both running and recovery periods for each participant before to each session. The athletes start their sprint by assuming a stationary posture, positioned posterior to a designated marker. Subsequently, the participants engaged in their HIIT session. During these training sessions, the athletes’ pace was determined by an examiner who emitted noises at regular intervals until the completion of the activity. During the 30-s active recovery persiod, athletes were required to traverse a distance that was determined based on their individual maximum aerobic velocity (MAV). The athletes refrained from engaging in running activities and instead assumed a stationary position while anticipating their subsequent exertion. During the interval of recuperation, a prolonged auditory signal was emitted at the midpoint (15 s) to notify the athletes of the remaining duration till the conclusion of the recovery phase. Following the completion of the HIIT session, athletes engaged in a cooling down period lasting about 15 min. This involved engaging in low-intensity jogging and completing static stretching exercises. Two members of our academic supervised all HIIT sessions (Ben Abderrahman et al., 2013). The control group withdrew from engaging in any additional interval training program for the duration of the 7-week training session. Furthermore, it is worth noting that both the experimental and control groups diligently engaged in their technical and tactical basketball training throughout the evening sessions, as seen in Table 3.

**TABLE 2:** The experimental group’s training program.

	Training load	Intensity	Frequency
Week 1	2×(8 × 30 s) R = 5 min TL:600 ATU	100/50% MAV.	3 days/week
Week 2	2×(10 × 30 s) R = 5 min TL:800 ATU	110/50% MAV.	3 days/week
Week 3	2×(8 × 30 s) R = 5 min TL:640 ATU	110/50% MAV.	3 days/week
Week 4	2×(10 × 30 s) R = 5 min TL:800 ATU	110/50% MAV.	3 days/week
Week 5	2×(10 × 30 s) R = 5 min TL:800 ATU	110/50% MAV.	3 days/week
Week 6	2×(10 × 30 s) R = 5 min TL:750 ATU	100/50% MAV.	3 days/week
Week 7	2×(10 × 30 s) R = 5 min TL:750 ATU	100/50% MAV.	3 days/week

MAV, maximal aerobic velocity; R, passive recovery between series; TL, training load; ATU, arbitrary training units Example: [2 x (8 × 30 s) 100/50% MAV. R = 5 min] it means that the subject had to run two series of eight times 30 s composed of 30 s running at 100% of MAV, and 30 s active recovery at 50% of MAV. The subject recovers passively 5 min between each series. Each session is repeated 3 times a week Example of training load calculation for the first week: [[(100 + 50)/2] x 4 × 2] = 600 ATU.

**TABLE 3:** Basketball tranining programme.

Week	Day	Content
1–2	Monday, Wednesday, Friday	✓ Dribble (crossover, between thelegs, behind the back, spin move, and inside/out)
		✓ Shoot off the dribble (cross over, between the legs, behind the back, spin move and inside/out)
		✓ Two-player sliding pass (chest pass, bounce pass, one hand pass with one and two ball)
		✓ Three-player moving pass (chest pass, bounce pass, one hand pass)
3–4		✓ Individual defense (sliding, side away running, slide, run slide, over play and stop the ball)
		✓ Two, three and four player fast break
		✓ Two and three player group cooperation
		✓ Team offense drills-offensive move to attack man to man defense
5–6		✓ Two and three player man to man defense (strong and weak side help and recovery concept)
		✓ Five-player fast break
		✓ Half-court three player group offensive and defensive drills
		✓ Team offense drills (offensive move to attack man to man defense)
7		✓ Half-court zone defense (2-3, 3-2, 1-1-3, 1-3-1)
		✓ Full-court zone defense (double-team): (2-2-1, 1-2-1-1)
		✓ Three, four, and five player fast break
		✓ Team offense drills-offensive move to attack zone defense

### Data analysis

Using G*Power 3.1.6, a power analysis (priori) was carried out for an experimental design in order to establish the sample size that should be used. Based on the effect sizes reported in experimental studies, the analysis indicated that minimally 27 participants for an α of 0.05 and a power of 0.90 would be required. SPSS 23.0 was used to analyze the data mean values, standard deviation of the mean, and mean differences (Chicago, IL, USA). The Shapiro-Wilk test was used to ensure that the parameters had a normal distribution and were homogeneous. After analyzing the athletes’ data, a *p*-value of 0.05 was determined to be significant for the difference between the pre- and post-test results. To test the differences between groups and compare the effects of pre and post-intervention, a two-way ANOVA for repeated measures was used. When *p* < 0.05 was found, the Bonferroni *post hoc* test was used to identify differences between groups while controlling for multiple comparisons. To compare within-group changes (pre to post-intervention), the power of the effect was calculated using partial eta squared (*η* ^*2*^) for ANOVA repeated measures. Partial eta squared with 0-0.06, 0.06-0.14, and >0.14 was classified as having a small, medium, or large effect, respectively (Stevens, 2012).

## Results

The data collected from the athletes at the end of the study is examined and given in tables below.

Cortisol, glucose, insulin, T4, and T3 concentrations as a result of the analysis of the samples (Mean ± SD) are shown in the Table 4. Cortisol, TSH, ACTH, and PTH concentrations were significantly in favor of the E.G., and the effect of training was moderate to high (*p* = 0.000). Furthermore, glucose, Insulin, T3, and T4 values were also in favor of the E.G., (*p* = 0.000). It has been determined that athletes control metabolic stress and affect their energy use levels more positively.

**TABLE 4:** Comparison of the control and experimental groups’ pre/post test parameters within and between groups.

		E.G (N = 14)			K.G (N = 13)		
Parameter’s		X ± Sd	*md*	*p*	X ± Sd	*md*	*p*
Cortisol (µg/dL)	Pre	10.37 ± 2.22	−0.73	0.205	10.79 ± 3.78	0.47	0.649
	Post	9.64 ± 2.26			11.26 ± 3.79		
T4 (ng/dL)	Pre	1.17 ± .14	0.03	0.881	1.32 ± .29	−0.04	0.462
	Post	1.20 ± .12			1.28 ± 0.08		
T3 (ng/dL)	Pre	2.83 ± .36	−0.02	0.090	3.16 ± .62	−0.08	0.710
	Post	2.81 ± .32			3.08 ± 0.27		
Insulin (mg/dL)	Pre	15.32 ± 6.52	0.33	0.962	22.90 ± 19.18	−0.61	0.871
	Post	15.65 ± 10.44			22.29 ± 8.20		
TSH (mU/mL)	Pre	2.36 ± 1.46	−0.39	0.000**	2.12 ± 0.31	0.11	0.246
	Post	1.97 ± 0.84			2.23 ± 0.74		
PTH (pg/ml)	Pre	44.14 ± 10	−10.56	0.010*	52.05 ± 13.39	−18.08	0.010*
	Post	33.58 ± 7.77			33.97 ± 5.38		
ACTH (pg/ml)	Pre	22.22 ± 6.14	8.04	0.001**	17.25 ± 2.60	14.00	0.000**
	Post	30.06 ± 12.13			31.25 ± 15.91		

*:*p* < 0.05; **:*p* < 0.001; md: mean differences p: comparison of intra-group pre-post test values.

When analyzing Table 5, it was seen that the post-test values of Glucose, Cholesterol, Triglyceride, HDL, and Mg showed statistical significance when compared to the pre-test values of the exercise group (*p* = 0.000). A comparable outcome is found in the overall effect of CG on Cholesterol and HDL levels, with a statistically significant *p*-value of 0.000. Findings from studies have shown that interval training has more efficacy, especially in relation to blood lipids.

**TABLE 5:** Comparison of the control and experimental groups’ pre/post test parameters within and between groups.

		E.G (N = 14)			K.G (N = 13)		
Parameter’s		X ± Sd	*md*	*p*	X ± Sd	*md*	*p*
Glucose (mg/dL)	Pre	86.44 ± 13.19	−2.81	0.865	86.45 ± 10.03	3.55	0.311
	Post	83.63 ± 10.70			90.00 ± 13.81		
Cholesterol (mg/dl)	Pre	150.39 ± 15.98	−7.83	0.000**	158.50 ± 31.33	−7.50	0.026*
	Post	142.56 ± 14.76			151.00 ± 16.59		
Triglyceride (mg/dl)	Pre	66.22 ± 24.84	−3.55	0.000**	65.75 ± 14.03	−1.37	0.350
	Post	62.67 ± 14.50			64.38 ± 14.66		
HDL (mg/dl)	Pre	61.83 ± 13.35	−9.27	0.000**	57.25 ± 19.38	−7.25	0.030*
	Post	52.56 ± 14.76			50.00 ± 4.69		
LDL (mg/dl)	Pre	74.17 ± 12.28	−0.67	0.440	77.63 ± 6.14	−0.62	0.455
	Post	73.50 ± 11.60			77.01 ± 27.45		
Albumin (g/dl)	Pre	4.69 ± 0.25	0.13	0.361	4.82 ± 0.52	0.22	0.801
	Post	4.82 ± 0.20			5.04 ± 0.16		
Ca (mg/dl)	Pre	10.20 ± 0.46	0.20	0.523	9.74 ± 0.45	0.41	0.367
	Post	10.46 ± 0.27			10.15 ± 0.25		
Mg (mg/dl)	Pre	1.76 **±** 0.06	0.03	0.045*	1.84 ± 0.11	0.08	0.260
	Post	1.79 ± 0.08			1.92 ± 0.17		

*:*p* < 0.05; **:*p* < 0.001; md: mean differences p: comparison of intra-group pre-post test values.

## Discussion

The aim of the study was to examine the effect of interval training on pituitary function levels in basketball players. The study was planned with the aim of comparing long-term consequences. Interval training has been shown to induce changes in pituitary function, as seen by study findings. The findings presented in our study indicate a considerable change in interval training subsequent to exercising. Furthermore, it was determined that the control group did not exhibit any statistically significant changes. In addition, it has been demonstrated that the post-test results of the interval training group presented greater significance and favored the exercise group over the control group.

**FIGURE 1:**
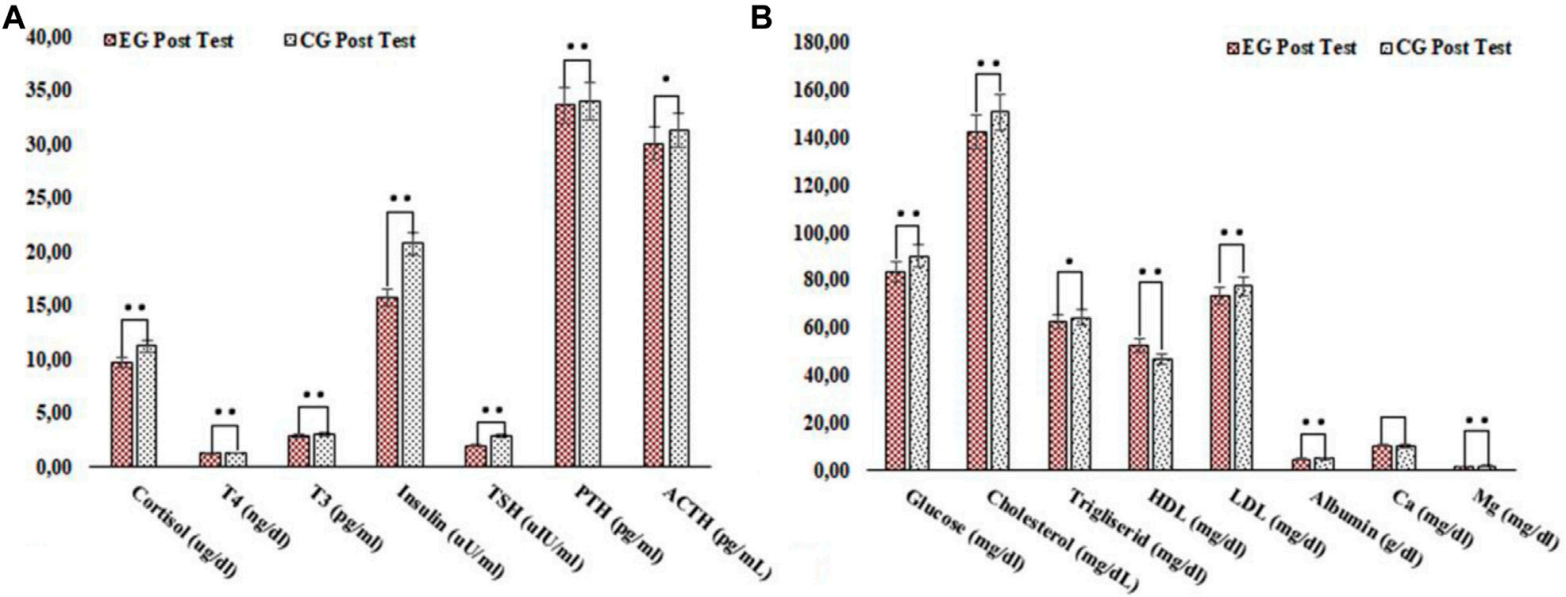
Comparison of the control and experimental groups’ post test parameters between groups. •:*p* < 0.05; ••:*p* < 0.001; p: comparison of the post-test values of the groups; η^2^: effect size: 0.10 small, 0.25 medium, and 0.40 large **(A)** Comparison of the control and experimental groups’ post test hormone parameters between groups **(B)** Comparison of the control and experimental groups’ post test blood glucose, lipid and mineral parameters between groups.

It might be hypothesized that the rationale for the occurrence comes in the advantageous impact of interval training on pituitary function. Although different training methods have been investigated in previous studies, we discuss the impact of exercise on both athletes and sedentary individuals separately to gain a deeper understanding of its chronic effects. Athletes are subjected to prolonged physical stress as a result of rigorous training and competitions. Several studies have linked this disorder to increased cortisol secretion (McEwen, 2003; Chrousos, 2009). Minetto et al. (2008) discovered that increasing training load resulted in higher levels of cortisol in athletes, which was positively correlated with performance (Minetto et al., 2008). According to Foley et al. (2008) aerobic training resulted in a statistically insignificant decrease in Cortisol levels after the sixth and 12th weeks (Foley et al., 2008). Furthermore, Skoluda et al. (2012) reported that cortisol levels in endurance athletes reached higher glucocorticoid levels than the control group (Skoluda et al., 2012). Exercise raises the level of cortisol in the blood and saliva, according to research, confirming previous findings (Gomez-Merino et al., 2006; Kraemer et al., 2008; Skoluda et al., 2012). Another study found a significant decrease in cortisol levels in athletes after long-term endurance exercise (Daly et al., 2005). Several studies in adults have suggested that plasma cortisol levels rise after 15–20 min of intense short-term exercise, but not immediately, but Klentrou et al. (2016) discovered that cortisol levels rose after 45 min of plyometric exercise, which they interpreted as an adrenocortical response to the duration and intensity of exercise (Klentrou et al., 2016). The decrease in cortisol after exercise is most likely due to an increase in cortisol removal from the circulation or a decrease in adrenocorticotropic hormone (Heshmat et al., 2010). Cortisol and ACTH levels increased with training duration and intensity, as expected from the literatüre (Figure 1; Table 3, 4). Cortisol levels were also significantly reduced after interval training. Cortisol levels decreased significantly during the preparation period, but increased before the competition, according to Hejazi and Hosseini’s study (Hejazi and Hosseini, 2012). Cortisol levels are higher during the pre-competition period due to the release of anabolic and catabolic hormones (Majumdar et al., 2010). It has been discovered that the amount of cortisol released throughout the day varies, with the most release occurring in the morning hours and the amount not increasing significantly as the afternoon approaches (Kakooei et al., 2009). Changes in cortisol levels measured using various methods may be an indication of this.

T3 and T4 hormones are secreted by the thyroid gland and are known to regulate general metabolism, growth, tissue differentiation, fatty acid oxidation, and thermoregulation (Alvero-Cruz et al., 2011). According to the literature, the levels of activity of these hormones vary. Researchers believe that these differences are influenced by factors such as the subjects’ physical condition, the intensity, duration, type, and age-sex differences, as well as the temperature of the environment (Ylli and Wartofsky, 2019). However, some studies have shown that T3 and T4 hormone levels increase with the intensity of aerobic exercise (Akgül and Baydil, 2021), while others have reported that T3 and T4 hormone levels do not increase acutely during exercise (Roa Dueñas et al., 2022). Similarly, in our study, we discovered that interval training has a significant and high impact on T3 and T4 values (Figure 1; Table 3; Table 4). Many studies have found that rowers with intermediate and advanced training have lower resting T3 and T4 hormone levels (Hooper et al., 2017; Kasper et al., 2019; Pedlar et al., 2019). In contrast, McMurray and Hackney’s (2005) study found that short-term exercise at 60% of VO2max increased TSH levels. However, increased TSH levels had no effect on T3, and T4 concentrations (McMurray and Hackney, 2005). Thyroid hormones are known to increase glucose absorption from the intestine and stimulate glycolysis by influencing carbohydrate conversion (Al-Bayati and Al-Khateeb, 2021). T3 indirectly affects hepatic glucose production and increases muscle glucose consumption in addition to increasing sensitization (Salvatore et al., 2014). Furthermore, thyroid hormones, which increase pancreatic cell sensitivity to stimuli that cause insulin release, play an active role in insulin secretion (Eom et al., 2022). In our study, glucose and insulin levels were significantly different from the control group. According to a literature review, weight changes can have a significant impact on the outcome of exercise studies (Ratamess, 2012; Hjorth et al., 2017; Ritz et al., 2019; Magkos, et al., 2020). Insulin resistance is commonly associated with an increase in abdominal fat and a decrease in muscle mass. This suggests that weight-loss programs aimed at reducing central body fat may be effective at lowering insulin resistance. Although weight loss was significant in the exercise group in the current study, the difference in insulin value between groups was insignificant. Teixeira de Lemos et al. (2012) reviewed the effects of regular exercise and concluded that regular exercise reduces total, visceral, and subcutaneous fat, controls glycemia, and increases free fatty acid oxidation without resulting in weight loss. According to Oberbach et al. (2006), combining endurance and aerobic exercise can result in a reduction in BMI. Some studies have found that exercise helps with weight loss, fasting glucose plasma levels, and insulin serum levels (Carroll and Dudfield, 2004; Church, 2011). Furthermore, it has been shown to be significantly related to increased insulin sensitivity, changes in body fat, and anti-inflammatory factors (Weyer et al., 2010; Apovian et al., 2015; Khera et al., 2016). As a result, it is thought that lifestyle changes, in addition to the training protocol used in our study, could result in earlier improvement in glycemia and insulin levels.

There are some limitations to our study that should be mentioned. Our subjects were chronically trained male basketball players who had adequate exercise experience. Given this, we would like to emphasize that our findings may not be applicable to females or recreational athletes, whose physiological responses may differ depending on their training regimen. Furthermore, comparisons with other athletes should be made with caution because their levels of training and physical preparation may differ. Athletes with endocrinological findings associated with insulin resistance should be evaluated differently than the rest of the sample. Despite its shortcomings, the study has several strengths. The study employed an experimental design. The findings of this study show that longitudinal training is beneficial to this sample group.

## Practical applications

The utilization of HIIT is frequently advocated by trainers and strength coaches as a means to enhance endurance performance. The findings of our study indicate that the training method known as HIIT does not have a significant influence on the extent of respiratory capacity enhancement. The findings of this study demonstrate the change in pituitary functions observed in two distinct groups: one engaged solely in HIIT, and the other focused exclusively on technical and tactical training. These changes were monitored over a period of seven weeks. If the objective of the HIIT program is to enhance endurance and optimize pituitary function within an improved reference range, it demonstrates the efficacy of employing the HIIT training approach. It is important to note that these results may exhibit variations in cases when training programs extend beyond an 8-week duration and for athletes having a limited exercise background. The findings of our study indicate that the HIIT training program leads to an elevation in the normal physiological stress on pituitary function. However, it is worth noting that this impact is comparatively more favorable when compared to the control group. It is important to acknowledge that these improvements may not necessarily result in significant enhancements in performance.

## Conclusion

Finally, short high-interval training protocol of three workouts per week for 7 weeks was shown to be effective in lowering cortisol levels in basketball players. Several factors may influence the effect of exercise on blood glucose and insulin levels in basketball players. Factors such as the exercise program, population differences, lifestyle habits, and baseline laboratory parameters all have an impact on the study’s results. As a result, it is recommended that all of the above factors be considered in order to achieve the desired level of cortisol and other parameters.

## Data Availability

The original contributions presented in the study are included in the article/Supplementary Material, further inquiries can be directed to the corresponding author.
